# Physicians' Perception of Telemedicine Use for Patients With Autism Spectrum Disorder at the Mental Health Hospital in Jeddah, Saudi Arabia: A Cross-Sectional Study

**DOI:** 10.7759/cureus.91125

**Published:** 2025-08-27

**Authors:** Ghada T Kersh, Abeer A Subke, Mohammed Alshehri

**Affiliations:** 1 Preventive Medicine, Ministry of Health, Jeddah, SAU; 2 Preventive Medicine Postgraduate Program, Ministry of Health, Jeddah, SAU; 3 Public Health Administration, Ministry of Health, Jeddah, SAU

**Keywords:** autism spectrum disorder, mental health, physicians' perception, saudi arabia, telemedicine

## Abstract

Background: Autism spectrum disorder (ASD) is a complex neurodevelopmental condition characterized by difficulties in social interaction, communication, and the presence of repetitive behaviors. The increasing prevalence of ASD highlights the critical need for early diagnosis and tailored, long-term management.

Objective: This study aims to assess the expectations, perceived benefits, and barriers related to telemedicine among physicians and psychiatric residents at the Mental Health Hospital.

Methods: A cross-sectional study was conducted at the Mental Health Hospital in Jeddah, Saudi Arabia, including all affiliated physicians and psychiatric residents through total population sampling. Data were collected using a secure, self-administered online questionnaire, which incorporated the validated Telemedicine Expectations Questionnaire (TEQ) to assess expectations, perceived benefits, and barriers to telemedicine. Appropriate descriptive and inferential statistics were used, with a p-value <0.05 considered statistically significant.

Results: The study involved 49 physicians aged 21-30, with a nearly equal gender distribution. Junior residents were the largest professional group at 34.69% (n=17). More than half of the participants agreed that telemedicine is a useful tool for diagnosing ASD (n=25, 51.02%) and for communicating diagnoses to families (n=22, 44.90%). However, fewer participants supported its use for treatment (n=17, 34.69%) or as a routine diagnostic tool (n=15, 30.61%). The most recognized benefits included improving parenting skills (n=29, 59.18%), saving time (n=22, 44.90%), and increasing care flexibility (n=24, 48.98%). Reported barriers involved poor internet connection (n=25, 51.02%), difficulty tracking children during sessions (n=20, 40.82%), and the child's embarrassment (n=29, 59.18%). No significant associations were found except for benefits, which varied by experience (p=0.037).

Conclusion: Telemedicine has become a viable alternative to traditional treatments, especially during public health crises like the COVID-19 pandemic, which disrupted in-person care. Despite challenges such as limited internet access, tele-assessments align closely with conventional evaluations, and parent-mediated tele-interventions have been shown to improve communication and social skills.

## Introduction

Autism spectrum disorder (ASD) is a neurodevelopmental disorder characterized by impairments in social interaction, communication difficulties, and restricted, repetitive behaviors [1]. The causes of ASD are not fully understood, but it is widely accepted that a combination of genetic, environmental, and prenatal factors contributes to its development. These factors can interact in complex ways, influencing neurodevelopment and potentially increasing the risk of ASD [2-5]. Environmental factors play a significant role in autism by influencing neurodevelopment and gene regulation [6]. Some cases are linked to specific genetic syndromes or identifiable environmental triggers, while others remain idiopathic with no clear cause. Factors such as heavy metal exposure, maternal infection, and folic acid supplementation have been associated with autism, either directly or through gene-environment interactions [7]. The global median prevalence of ASD was estimated to be 1%, with Europe showing a range of 0.24%-2.68% [8]. Furthermore, a study conducted in Riyadh, Saudi Arabia, focused on children aged 2-4 and found that the prevalence of autism was 2.51%, translating to 25 cases per 1000 children [9].

Continuous management of ASD is crucial due to its lifelong nature and evolving needs across various life stages. Effective management requires ongoing monitoring, assessment, and adjustments to treatment plans to address the disorder's complexity and variability [10-12]. An integrated care pathway, focusing on individualization, support, and continuous training, is essential for providing comprehensive and coordinated care [10]. Behavioral therapies and pharmaceutical interventions play a significant role in managing ASD symptoms, with medications such as antidepressants, stimulants, and antipsychotics targeting comorbidities and core symptoms, especially in older individuals [13,14]. Various global intervention models focus on reducing symptoms and improving social skills through clinical, scientific, psychological, family-based, and rehabilitation strategies [15]. However, conventional in-person ASD care faces significant limitations due to geographical barriers, long waiting times for appointments, and a lack of specialized professionals.

Telemedicine has emerged as a valuable tool in the screening, assessment, and diagnosis of ASD, offering benefits such as reduced waiting times and increased accessibility to healthcare services for patients with ASD and their families [16]. The COVID-19 pandemic accelerated the adoption of telemedicine in ASD services, as social restrictions and lockdowns necessitated innovative remote care solutions to maintain diagnosis, treatment, and support. Studies have shown that tele-assessment for ASD in toddlers can achieve high diagnostic agreement with traditional in-person assessments, indicating its reliability and acceptability among clinicians and families [17]. Furthermore, parent-mediated telehealth interventions have demonstrated promising outcomes in improving social skills, communication abilities, and cognitive abilities in children with ASD, especially during the COVID-19 pandemic, when in-person interventions may be challenging [18]. Understanding the expectations and concerns of both healthcare professionals and parents regarding telemedicine for ASD is essential to tailor interventions effectively and address perceived barriers for successful implementation and improved patient outcomes [19]. Incorporating telemedicine into the care of ASD patients can enhance access to timely and effective interventions, ultimately improving the quality of life for individuals with ASD and their families.

Despite its potential, telemedicine in ASD care faces several challenges. Common barriers include limited access to reliable devices and internet connectivity, healthcare professionals’ unfamiliarity with telehealth technologies, and difficulties adapting assessments to virtual formats. Additionally, logistical issues and unclear expectations can affect the usability of remote visits for individuals with ASD, especially considering the disorder’s varying severity and complexity. Addressing these obstacles is essential to optimize telemedicine’s effectiveness and ensure it meets the unique needs of patients and their families.

Physician acceptance and engagement are crucial for the successful integration of telemedicine into clinical practice. Assessing physicians' perceptions regarding telemedicine is therefore important, as their views directly influence the adoption and effectiveness of this technology. Research on telemedicine for ASD remains limited, despite growing interest in its potential benefits and associated challenges. Understanding physicians' perceptions and the perspectives of specialists is essential for developing effective strategies to implement telemedicine in psychiatric care for individuals with ASD. This study aims to assess physicians’ attitudes toward telemedicine use for ASD patients at the Mental Health Hospital in Jeddah, Saudi Arabia, and to identify the perceived barriers to its implementation.

## Materials and methods

Study design and population

This is a cross-sectional study that was conducted from October 2024 to April 2025 at the Mental Health Hospital in Jeddah, Saudi Arabia. The study population comprised all physicians affiliated with the hospital and all psychiatric residents (both junior and senior levels) who were part of the hospital’s psychiatric training program.

Sampling and sample size

The sampling technique involved obtaining a list of all physicians and residents, including their contact details. This list served as the sampling frame. Given the estimated population size of 50 individuals, with a desired 5% margin of error and 95% confidence level, the study aimed to recruit the entire population to ensure comprehensive coverage and maximize statistical power.

Data sources and collection

The study data were collected through a self-administered questionnaire created electronically using a secure online survey platform. The questionnaire was sent to participants through their official email addresses, as registered with the administration office at the Mental Health Hospital in Jeddah, with clear instructions on completing it at their convenience. To maximize participation rates and to avoid non-response bias, follow-up reminders were delivered via official emails. The participants’ socio-demographic data, including age, gender, and residency level, were collected in the first section. In the second and third sections, we used the Telemedicine Expectations Questionnaire (TEQ), developed by Gabellone et al. [17]. The TEQ is based on several published articles and demonstrates strong internal consistency (Cronbach's α of 0.95).

Expectations about the uses and potential benefits of telemedicine were assessed through 16 items, divided into two distinct subscales: (I) Uses and Willingness to Use Telemedicine and (II) Perceived Potential Benefits of Telemedicine. All items were measured using a five-point Likert scale, where "strongly agree" = 1 and "strongly disagree" = 5, with higher scores indicating greater disagreement. The Uses and Willingness to Use Telemedicine subscale (Items a-h) consisted of eight items evaluating participants’ beliefs regarding the utility of telemedicine for diagnosing and treating ASD, its integration into traditional care, and their willingness to adopt it in routine or emergency settings. The Perceived Potential Benefits subscale (Items i-p) included eight items assessing views on the advantages of telemedicine, such as cost and time savings, enhanced family routine management, service delivery flexibility, and the opportunity to observe the child in a home environment. For each participant, subscale scores were calculated by summing responses to the respective items. The total score for each subscale ranged from 8 to 40.

The third section included two multi-option checklists consisting of objective barriers (six items) and subjective barriers (seven items) to telemedicine. Participants rated their level of agreement with barrier-related statements using a three-point Likert scale (agree, neutral, and disagree). These responses were analyzed descriptively to explore overall concerns regarding telemedicine implementation.

Total scores for each domain were categorized into three agreement levels based on sample percentiles: high agreement (≤25th percentile), moderate agreement (26th-75th percentile), and low agreement (≥76th percentile). Specifically, for the use and willingness domain, scores ≤18 indicated high agreement, 19-24 moderate agreement, and >24 low agreement. For the perceived potential benefits domain, scores ≤16 corresponded to high agreement, 17-21 to moderate agreement, and >21 to low agreement. For the perceived barriers domain, scores ≤19 represented high agreement, 20-25 moderate agreement, and >25 low agreement.

Statistical analysis

All data were analyzed using IBM SPSS Statistics for Windows, Version 25.0 (Released 2017; IBM Corp., Armonk, New York, United States). For each participant, subscale scores were calculated by adding responses to the respective items. Descriptive statistics were computed for continuous variables and reported as mean ± standard deviation (SD) and median with interquartile range (IQR), while categorical variables were expressed as frequencies and percentages. The mean score for each domain was computed by calculating the average of all respondents’ scores for that item, reflecting the overall level of agreement or disagreement. The median score was determined by identifying the midpoint value in the ordered distribution of responses for each item, providing a robust measure of central tendency. These descriptive statistics were used to evaluate general trends in participants' responses across the various TEQ domains. Prior to inferential analysis, the distribution of total scores for each domain (use and willingness, perceived potential benefit, and barrier factors) was assessed using the Shapiro-Wilk test, which indicated non-normal distribution. Accordingly, non-parametric tests were applied. The Mann-Whitney U test was used to compare differences in median scores between two groups, and the Kruskal-Wallis test was employed for comparisons involving more than two groups. Additionally, to examine the independent association between demographic variables and each of the three outcome domains while adjusting for potential confounders, a general linear model (GLM) was applied. Despite the non-normality of the outcome variables, GLM was used given its robustness to violations of normality to allow the assessment of multiple predictors simultaneously. A p-value of <0.05 was considered statistically significant. All statistical tests were two-tailed.

## Results

A total of 49 physicians participated in the study assessing perceptions of telemedicine use for patients with ASD in Saudi Arabia. The majority of participants were aged between 21 and 30 years (57.14%), followed by those aged 31-40 years (30.61%), and a smaller proportion aged 41-51 years (12.24%). The gender distribution was nearly equal, with 51.02% female and 48.89% male participants. Regarding professional level, junior residents constituted the largest group (34.69%), followed by senior residents (28.57%), senior registrars (18.37%), specialists (12.24%), and consultants (6.12%) (Table 1).

**Table 1:** Demographic characteristics of study participants.

Variable	Frequency, N (%)
Age	
21-30	28 (57.14)
31-40	15 (30.61)
41-51	6 (12.24)
Gender	
Male	24 (48.89)
Female	25 (51.02)
Current level of training or position	
Junior resident	17 (34.69)
Senior resident	14 (28.57)
Senior registrar	9 (18.37)
Specialist	6 (12.24)
Consultant	3 (6.12)

Over half of the participants agreed that telemedicine is a useful tool for diagnosing (51.02%) and communicating diagnoses or recommendations to families (44.90%). However, fewer participants expressed strong agreement regarding its use for treatment (34.69%) or as a routine tool for ASD diagnosis (30.6%), with a notable proportion remaining neutral or disagreeing. Notably, 40.82% of respondents agreed they would use telemedicine in emergencies, reflecting a cautious but situationally receptive stance.

Perceived benefits of telemedicine were well acknowledged, particularly for improving parenting skills (59.18%), saving time (44.90%), and increasing care flexibility (48.98%). Over half of the respondents (53.06%) supported its role in observing children in their natural home environment (Table 2).

**Table 2:** Participants' level of agreement on use and willingness and potential benefits. ASD: autism spectrum disorder.

		Strongly agree N (%)	Agree N (%)	Neutral N (%)	Disagree N (%)	Strongly disagree N (%)	Mean ± SD	Median (IQR)
Use and willingness	a. Telemedicine is a useful tool for diagnosing ASD	3 (6.12)	25 (51.02)	9 (18.37)	8 (16.33)	4 (8.16)		
	b. Telemedicine is a useful tool for treating ASD	2 (4.08)	17 (34.69)	19 (38.78)	9 (18.37)	2 (4.08)		
	c. Telemedicine is a useful tool for communicating diagnoses or providing recommendations to families	7 (14.29)	22 (44.90)	14 (28.57)	6 (12.24)			
	d. Telemedicine is a useful integration into traditional face-to-face diagnosis	5 (10.20)	14 (28.57)	18 (36.73)	11 (22.45)	1 (2.04)		
	e. Telemedicine is a useful integration into traditional face-to-face treatment	4 (8.16)	24 (48.98)	14 (28.57)	5 (10.20)	2 (4.08)		
	f. I am willing to use telemedicine as a routine tool for the diagnosis of ASD	3 (6.12)	15 (30.61)	17 (34.69)	8 (16.33)	6 (12.24)		
	g. I am willing to use telemedicine as a routine tool for the treatment of ASD	5 (10.20)	18 (36.73)	15 (30.61)	7 (!4.29)	4 (8.16)		
	h. I am willing to use telemedicine for the diagnosis and treatment of ASD only in emergency situations	7 (14.29)	20 (40.82)	11 (22.45)	7 (14.29)	4 (8.16)		
	Total a–h						21.55 ± 5.75	21 (18-24)
Potential benefits	i. Telemedicine is a useful tool for improving parenting skills in managing behavioural problems in children and adolescents with ASD	6 (12.24)	29 (59.18)	13 (26.53)	1 (2.04)	-		
	j. Telemedicine is a useful tool for reducing behavioural problems of children and adolescents with ASD at home	1 (2.04)	19 (38.78)	20 (40.82)	9 (18.37)	-		
	k. Telemedicine reduces costs for accessing care (e.g., travel time, transportation expenses, missed work)	10 (20.41)	21 (42.86)	14 (28.57)	4 (8.16)	-		
	l. Telemedicine saves time (waiting time in clinics, travel time to the center, etc.)	10 (20.41)	22 (44.90)	14 (28.57)	3 (6.12)	-		
	m. Telemedicine improves the management of family routine	5 (10.20)	25 (51.02)	13 (26.53)	6 (12.24)	-		
	n. Telemedicine increases flexibility in offering care services	10 (20.41)	24 (48.98)	13 (26.53)	2 (4.08)	-		
	o. Telemedicine allows both divorced parents to contribute to their child’s assessment or intervention	9 (18.37)	25 (51.02)	14 (28.57)	-	1 (2.04)		
	p. Telemedicine allows the ASD child to be observed in the home environment	7 (14.29)	26 (53.06)	13 (26.53)	2 (4.08)	1 (2.04)		
	Total i–p						18.37 ± 3.91	18 (16-21.5)

Key objective barriers include issues such as poor internet connection (51.02%) and lack of digital devices (34.69%), while challenges like the inability to track moving children with cameras (40.82%) were also noted. Subjectively, the severity of ASD (48.98%) and the child’s embarrassment during sessions (59.18%) were significant barriers. Additionally, distractions from digital devices and the home environment affecting compliance were identified by over half of the participants (Table 3).

**Table 3:** Perceived subjective and objective barrier factors affecting the use of telemedicine. ASD: autism spectrum disorder.

Objective barrier	Agree N (%)	Neutral N (%)	Disagree N (%)
a. Lack of digital devices	17 (34.69)	17 (34.69)	15 (30.61)
b. Lack of knowledge of digital technology	20 (40.82)	19 (38.78)	10 (20.41)
c. Poor quality internet connection	25 (51.02)	17 (34.69)	7 (14.29)
d. Active involvement/supervision of parents during telemedicine services	14 (28.57)	26 (53.06)	9 (18.37)
e. Presence of at-home distractions	17 (34.69)	25 (51.02)	7 (14.29)
f. Camera is unable to follow children as they moved around	20 (40.82)	25 (51.02)	4 (8.16)
Subjective barrier			
a. Severity level of ASD	24 (48.98)	22 (44.90)	3 (6.12)
b. ASD child’s embarrassment during telemedicine services	29 (59.18)	15 (30.61)	5 (10.20)
c. Changes in ASD child's behavior during telemedicine services	28 (57.14)	16 (32.65)	5 (10.20)
d. Distraction of ASD child due to the use of digital devices	24 (48.98)	22 (44.90)	3 (6.12)
e. ASD child’s compliance influenced by the home setting	25 (51.02)	19 (38.78)	5 (10.20)
f. Changes in the traditional doctor-patient relationship	20 (40.82)	25 (51.02)	4 (8.16)
g. Potential negative effects of digital devices	20 (40.82)	16 (32.65)	13 (26.53)

The non-parametric tests (Mann-Whitney U and Kruskal-Wallis H) were applied to compare the total scores across different sociodemographic variables due to the non-normal distribution of the outcome variables. The analysis (Table 4) revealed no statistically significant differences in the "use and willingness" scores across age groups (p=0.523), gender (p=0.321), training levels (p=0.138), or years of professional experience (p=0.641). Similarly, perceived barriers scores showed no significant variation across age (p=0.332), gender (p=0.594), training level (p=0.754), or years of experience (p=0.120). However, a statistically significant difference was observed in the "potential benefit" scores across years of professional experience (p=0.037). Participants with less than one year of experience had the lowest mean score (14.50 ± 3.66). In contrast, those with 6-10 years of experience had the highest mean score (20.38 ± 3.96), reflecting the lowest level of agreement. Participants with 1-5 years and more than 10 years of experience reported intermediate mean scores of 18.85 ± 3.50 and 18.71 ± 3.35, respectively. No other statistically significant differences were noted for the "potential benefit" scores across age (p=0.436), gender (p=0.651), or training level (p=0.668) (Table 4).

**Table 4:** Comparison of participants' characteristics across outcome domains. *Kruskal–Wallis H statistic (degrees of freedom): comparisons among more than two groups were analyzed using the Kruskal–Wallis test. ^†^Mann–Whitney U statistic: comparisons between two groups were analyzed using the Mann–Whitney U test.

			Mean ± SD	Median (IQR)	Test statistics	p value
	Age group					
Use and willingness		21–30	22.11 ± 5.70	22.00 (18.25–24.50)	1.296(2)^*^	0.523
		31–40	21.13 ± 6.66	20.00 (16.00–24.00)		
		41–51	20.00 ± 3.52	20.50 (16.00–23.50)		
Potential benefit		21–30	17.64 ± 4.09	17.50 (15.00–21.00)	1.662(2)^*^	0.436
		31–40	19.20 ± 3.41	19.00 (15.60–21.00)		
		41–51	19.67 ± 4.13	19.50 (15.75–24.00)		
Perceived barriers		21–30	21.36 ± 4.23	23.00 (17.50–24.00)	2.205(2)^*^	0.332
		31–40	21.53 ± 3.25	22.00 (19.00–25.00)		
		41–51	23.67 ± 2.73	24.50 (21.21–26.00)		
Gender						
Use and willingness		Male	21.54 ± 6.30	20.00 (17.00–22.75)	250.5^†^	0.321
		Female	21.56 ± 5.29	22.00 (18.50–24.50)		
Potential benefit		Male	17.96 ± 3.72	17.50 (16.00–21.00)	277.5^†^	0.651
		Female	18.76 ± 4.13	20.00 (15.00–22.00)		
Perceived barriers		Male	21.96 ± 3.70	23.00 (19.50–25.00)	273.5^†^	0.594
		Female	21.44 ± 3.96	23.00 (19.00–24.00)		
Training level/position						
Use and willingness		Junior resident	20.47 ± 5.35	20.00 (17.50–22.50)	6.952(4)^*^	0.138
		Senior resident	24.71 ± 6.62	22.00 (20.75–29.50)		
		Senior registrar	21.33 ± 4.39	20.00 (17.50–24.00)		
		Specialist	18.00 ± 5.14	17.50 (14.25–21.00)		
		Consultant	20.67 ± 4.16	22.00 (16.00–22.00)		
Potential benefit		Junior Resident	17.12 ± 4.47	17.00 (12.00–21.50)	2.372(4)^*^	0.668
		Senior Resident	18.36 ± 3.23	18.00 (15.75–21.00)		
		Senior registrar	19.78 ± 4.02	19.00 (16.50–23.00)		
		Specialist	18.83 ± 3.13	18.50 (16.00–21.00)		
		Consultant	20.33 ± 4.73	22.00 (18.50–23.00)		
Perceived barriers		Junior resident	21.41 ± 4.06	23.00 (18.00–24.50)	1.902(4)^*^	0.754
		Senior resident	21.64 ± 4.29	23.00 (19.50–24.25)		
		Senior registrar	21.33 ± 3.67	22.00 (19.50–24.50)		
		Specialist	21.83 ± 3.19	22.00 (18.75–25.00)		
		Consultant	24.33 ± 2.08	25.00 (23.50–25.50)		
Years of experience						
Use and willingness		<1 year	19.63 ± 6.39	19.50 (17.25–22.00)	1.682(3)^*^	0.641
		1–5 years	22.77 ± 5.89	21.50 (18.00–25.25)		
		6–10 years	20.38 ± 6.21	19.50 (15.25–26.25)		
		>10 years	20.57 ± 3.55	22.00 (16.00–24.00)		
Potential benefit		<1 year	14.50 ± 3.66	14.50 (11.25–16.75)	8.491(3)^*^	0.037
		1–5 years	18.85 ± 3.50	19.00 (16.00–22.00)		
		6–10 years	20.38 ± 3.96	20.50 (16.25–23.50)		
		>10 years	18.71 ± 3.35	17.00 (16.00–22.00)		
Perceived barriers		<1 year	21.88 ± 3.04	21.50 (19.50–24.75)	5.832(3)^*^	0.120
		1–5 years	21.58 ± 4.23	23.00 (20.00–24.00)		
		6–10 years	19.88 ± 3.36	19.50 (16.50–23.25)		
		>10 years	24.00 ± 2.58	25.00 (22.00–26.00)		

**Figure 1:**
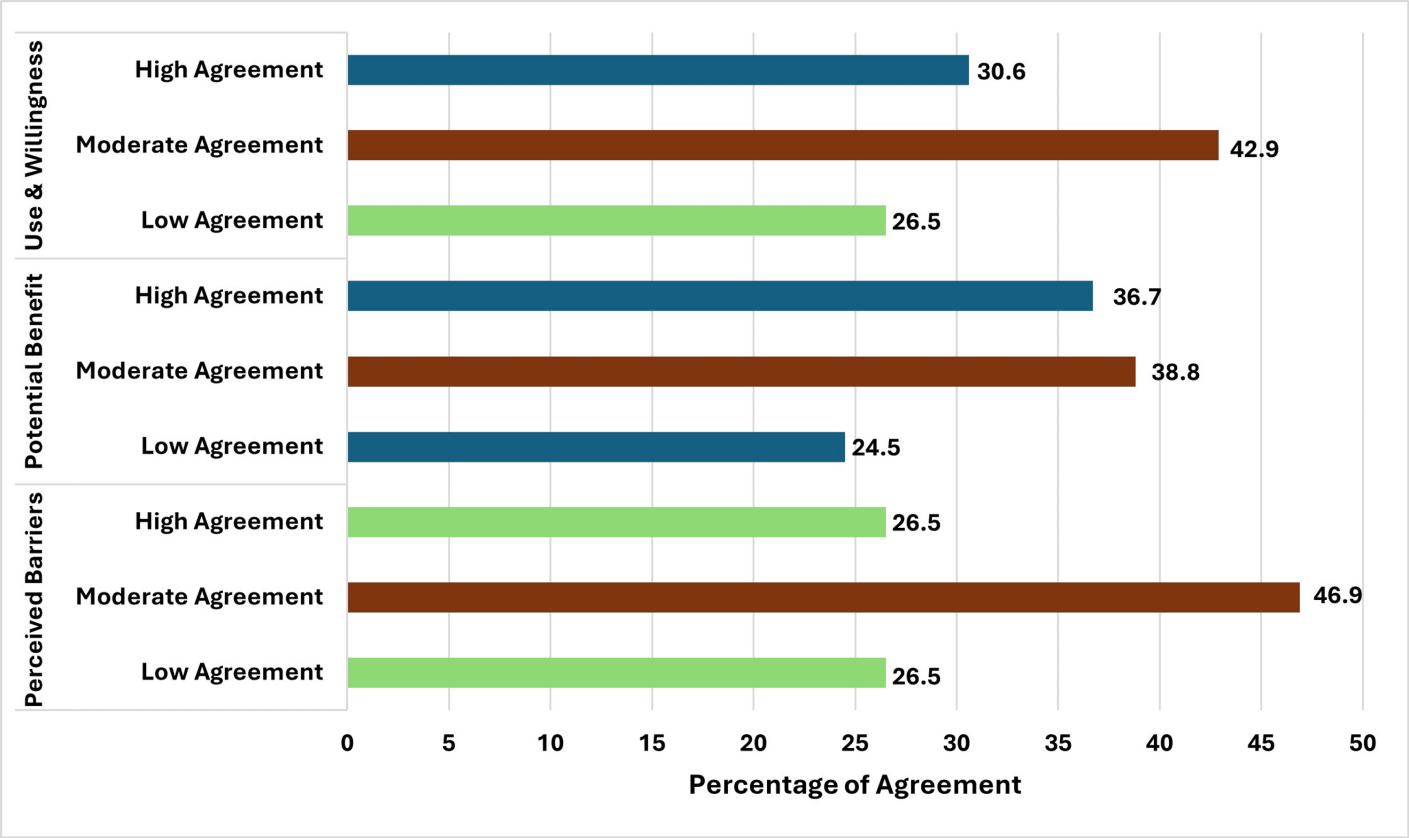
Distribution of participants’ agreement levels on use & willingness, potential benefits, and perceived barriers of telemedicine for ASD care. ASD: autism spectrum disorder.

The descriptive statistics of agreement levels across different domains (use and willingness, potential benefit, and perceived barriers) reveal notable trends. In all three domains, the majority of participants fell within the moderate agreement category, indicating a general but not overwhelming level of acceptance. The highest proportion of high agreement (36.7%) was observed in the potential benefit category, suggesting that participants perceive significant advantages. Meanwhile, use and willingness showed the highest proportion (30.6%) in the high agreement category, while perceived barriers had the lowest (26.5%). Notably, the perceived barriers category had the largest moderate agreement group (46.9%). The distribution of low agreement responses remained relatively similar across domains, with potential benefit (24.5%) showing slightly lower skepticism than use and willingness (26.5%) and perceived barriers (26.5%) (Table 5 and Figure 1).

**Table 5:** Participants' overall level of agreement on use and willingness, potential benefits, and perceived barriers. Agreement levels were categorized based on total scores using percentile-based cutoffs: high agreement (≤25th percentile), moderate agreement (26th–75th percentile), and low agreement (≥76th percentile).  For the use and willingness domain, scores ≤18 indicated high agreement, 19–24 indicated moderate agreement, and >24 indicated low agreement. For the perceived potential benefits domain, scores ≤16 indicated high agreement, 17–21 indicated moderate agreement, and >21 indicated low agreement. For the perceived barriers domain, scores ≤19 indicated high agreement, 20–25 indicated moderate agreement, and >25 indicated low agreement.

Category	Use and willingness N (%)	Potential benefit N (%)	Perceived barriers N (%)
High agreement	15 (30.6%)	18 (36.7%)	13 (26.5%)
Moderate agreement	21 (42.9%)	19 (38.8%)	23 (46.9%)
Low agreement	13 (26.5%)	12 (24.5%)	13 (26.5%)

The GLM analysis examined the influence of demographic and professional variables on three outcome scores: use and willingness, perceived potential benefit, and perceived barriers. The results revealed that none of the examined predictors (age, gender, current level of training, or years of professional experience) were statistically significant in predicting any of the outcome scores (all p-values >0.05). While the model predicting perceived potential benefit showed a slightly higher explanatory power (R²=0.100), no individual predictor reached statistical significance. Effect sizes for all predictors were small. These findings suggest that the participants’ demographic and professional characteristics did not meaningfully contribute to differences in agreement (Table 6).

**Table 6:** GLM analysis of the association between demographic variables and outcome domains. GLM: general linear model; F: F-statistic from the GLM analysis; R²: regression coefficient.

Outcome variable	Predictor	F	p-value	Effect size (η²)	R²
Use and willingness score	Age	0.784	0.381	0.028	0.037
	Gender	0.102	0.751	0.045	
	Training level	0.094	0.761	0.185	
	Years of experience	0.908	0.346	0.112	
Potential benefit score	Age	0.096	0.759	0.001	0.100
	Gender	0.186	0.668	0.003	
	Training level	0.021	0.886	0.106	
	Years of experience	1.381	0.246	0.198	
Barrier/factor score	Age	0.589	0.447	0.044	0.032
	Gender	0.056	0.815	0.047	
	Training level	0.004	0.953	0.105	
	Years of experience	0.073	0.788	0.086	

## Discussion

This cross-sectional study aims to assess the perception of physicians regarding the use of telemedicine for ASD patients in Saudi Arabia. In all three domains, the majority of participants fell within the moderate agreement category, indicating a general, but not overwhelming, level of acceptance. The highest proportion of high agreement (36.7%) was observed in the potential benefit category, suggesting that participants perceive significant advantages. Meanwhile, use and willingness had 30.6% in the high agreement category, while perceived barriers had the lowest at 26.5%, indicating that external factors may not be as strongly endorsed. Notably, the perceived barriers category had the largest moderate agreement group (46.9%), reflecting mixed opinions on external influences. The distribution of low agreement responses remained relatively similar across domains, with potential benefit (24.5%) showing slightly lower skepticism than use and willingness (26.5%) and perceived barriers (26.5%). These findings indicate that professional experience exhibited an inverse relationship with the perceived potential benefits of telemedicine and showed a statistically significant difference in "potential benefit" scores across years of professional experience (p=0.037). Furthermore, no significant associations were identified in relation to gender, age, or level of training. Participants in the study identified some subjective and objective barriers to using telemedicine in ASD. These barriers include a lack of digital devices, the severity of ASD, the inability to track children with cameras in different environments, poor internet connection, and children's embarrassment during the session. These were the most significant barriers.

Findings from our study align with existing literature. The World Health Organization (WHO) highlighted the benefits of telemedicine, such as reducing waiting times in hospitals and healthcare institutes, overcoming the isolation of severely ill patients, decreasing the time chronically ill patients spend in hospitals, overcoming barriers of illiteracy by providing medical information in different media, providing health information and education to patients in rural areas, increasing the number of people diagnosed and treated at their local hospitals without the need to be referred to another hospital, enabling healthcare professionals in isolated and rural areas to receive trainings and exchange information and knowledge with other specialists, and reducing the transportation costs for patients [20]. However, in their study, Mulligan and Ayoub [21] discussed the benefits and barriers of using telemedicine in psychological assessment. They stated that the critical behavioral observation of how the child approaches the tasks, or their levels of frustration, can be lost through remote assessment, particularly in children, where remote assessments result in less consistent outcomes in comparison with children assessed in person. Additionally, Mulligan and Ayoub [21] reported that most studies conducted on using telemedicine to assess neuropsychological measures, regarding the diagnostic accuracy and diagnostic agreement, were confined to adults under 65 years of age. These studies reported homogeneous results between in-person assessment and remote assessment. However, studies that included children or adults over 75 obtained heterogeneous results between in-person and remote assessments. This supports our participants' hesitance to solely depend on telemedicine for diagnosis and treatment.

Ali et al. [22] reported similar findings during the COVID-19 pandemic in the UK. The participants in the study confirmed that using telemedicine was cost-effective and time-saving for the patients. However, they faced difficulties accessing the telehealth service due to digital poverty and poor internet connection, in addition to their concerns about patient privacy [22]. Additionally, the participants in their study reported similar results regarding concerns about the severity of ASD and the effectiveness of remote assessment [22]. In line with our study results, Kellom et al. [23] found high satisfaction rates among developmental-behavioral pediatricians (92.23%) and caregivers (73.7%). The participants in the study confirmed that using telemedicine to assess children with ASD was cost-effective and time-saving, reduced the anxiety children experienced during long in-person sessions, facilitated faster visits, and allowed observation in the child’s normal environment. Similar to the participants in our study and the previously mentioned studies, the participants in this study were concerned about digital poverty and privacy [23]. Further supporting evidence from Liu et al. [16] demonstrated promising sensitivity and specificity metrics for telemedicine diagnostic tools, such as the Cognoa and NODA applications, which reached 0.98 sensitivity and 0.79 specificity and 0.85 sensitivity and 0.94 specificity, respectively [16]. 

However, these telemedicine applications have several limitations. Telemedicine applications, which are used for diagnosing and screening ASD, have little to no studies proving their psychometric properties, reliability, and validity [16]. Gabellone et al. [17] added another dimension by surveying 45 parents and 50 healthcare professionals about the use of telemedicine in diagnosing, treating, and screening children and adolescents with ASD. The response of the two groups varied significantly. Parents had high expectations for the use of telemedicine with their ASD children, whereas healthcare professionals had several concerns about its use. In line with our study and the previously mentioned studies, healthcare professionals in this study were willing to use it but were also concerned about the accessibility of patients to digital equipment and poor internet connection. They also stated that ASD patients need to be observed in environments other than their home settings. Additionally, clinicians stated that telemedicine can be used in the treatment of ASD, but it will not provide an accurate diagnosis.

Conversely, parents viewed telemedicine as a tool that can improve their parenting skills in managing the behavioral disturbances of their children [17]. Our findings also resonate with Yosep et al. [19], who highlighted the effectiveness of parent-mediated interventions in managing children with ASD, which improved children’s compliance, social skills, intelligence, and communication skills, in addition to increasing the parents’ knowledge and satisfaction [19]. Telemedicine can support such interventions by providing parents with timely education and coaching. However, it cannot replace in-person clinical assessment and support, specifically in severe ASD cases.

Overall, telemedicine offers significant promise in enhancing access to care for children with ASD and their families by mitigating logistical barriers. However, rigorous research is essential to validate its reliability and efficacy in the diagnosis, screening, and treatment of ASD. Addressing digital inequities is vital; expanding internet infrastructure and improving access to devices are necessary for the equitable delivery of healthcare services. Furthermore, effective implementation requires that healthcare professionals, patients, and caregivers develop the requisite technological competencies and communication strategies to navigate telemedicine platforms successfully, thereby fostering meaningful engagement in virtual care environments.

Strengths and limitations

The strengths of this study are notable, particularly the use of a validated measurement instrument, which enhances the reliability of the findings. Additionally, the sample comprises a diverse array of participants across different levels of residency, contributing to the generalizability of the results within the psychiatric context. Furthermore, the analysis was underpinned by robust statistical methodologies, reinforcing the study's rigor. However, it is important to acknowledge some limitations, including a relatively small sample size (n=49) that may restrict the representativeness of the findings. Moreover, the exclusivity of the sample, consisting solely of psychiatric residents, may not encompass the perspectives of all healthcare professionals engaged in the care of individuals with ASD. The cross-sectional design further constrains the ability to draw causal inferences, while reliance on self-reported data introduces potential confounding factors, such as social desirability bias and recall bias, which could affect the accuracy of the responses provided.

## Conclusions

This study examines the perceptions of psychiatric residents in Saudi Arabia regarding the utility of telemedicine as a moderately valuable resource for the management of ASD. The findings indicate that telemedicine significantly enhances access to care, streamlines time management, and facilitates a family-centered approach to treatment. Despite these benefits, the widespread adoption of telemedicine is hampered by several critical barriers, including technological limitations, concerns regarding patient compliance, and varying levels of confidence among practitioners regarding the effectiveness of telemedicine interventions. To mitigate these challenges, targeted strategies should be implemented, such as comprehensive physician training programs, advancements in technological infrastructure, and robust patient education initiatives. Furthermore, future research endeavors should seek to broaden the scope to encompass additional medical specialties. Longitudinal studies could provide insightful data on how perceptions of telemedicine evolve over time as both practitioners and patients gain more exposure and experience with its utilization in clinical practice.
